# Antifungal Susceptibility in Serum and Virulence Determinants of *Candida* Bloodstream Isolates from Hong Kong

**DOI:** 10.3389/fmicb.2016.00216

**Published:** 2016-02-26

**Authors:** Chaminda J. Seneviratne, Suhasini Rajan, Sarah S. W. Wong, Dominic N. C. Tsang, Christopher K. C. Lai, Lakshman P. Samaranayake, Lijian Jin

**Affiliations:** ^1^Oral Sciences, Faculty of Dentistry, National University of SingaporeSingapore; ^2^Faculty of Dentistry, The University of Hong KongHong Kong, China; ^3^Department of Pathology, Queen Elizabeth HospitalHong Kong, China; ^4^School of Dentistry, University of QueenslandBrisbane, QLD, Australia

**Keywords:** *Candida*, antifungal susceptibility, virulence factors, clinical isolates, plasma protein binding

## Abstract

*Candida* bloodstream infections (CBI) are one of the most common nosocomial infections globally, and they account for a high mortality rate. The increasing global prevalence of drug-resistant *Candida* strains has also been posing a challenge to clinicians. In this study, we comprehensively evaluated the biofilm formation and production of hemolysin and proteinase of 63 CBI isolates derived from a hospital setting in Hong Kong as well as their antifungal susceptibility both in the presence and in the absence of human serum, using standard methodology. *Candida albicans* was the predominant species among the 63 CBI isolates collected, and non-*albicans Candida* species accounted for approximately one third of the isolates (36.5%). Of them, *Candida tropicalis* was the most common non-*albicans Candida* species. A high proportion (31.7%) of the CBI isolates (40% of *C. albicans* isolates, 10% of *C. tropicalis* isolates, 11% of *C. parapsilosis* isolates, and 100% of *C. glabrata* isolates) were found to be resistant to fluconazole. One of the isolates (*C. tropicalis*) was resistant to amphotericin B. A rising prevalence of drug-resistance CBI isolates in Hong Kong was observed with reference to a previous study. Notably, all non-*albicans Candida* species, showed increased hemolytic activity relative to *C. albicans*, whilst *C. albicans, C. tropicalis*, and *C. parapsilosis* exhibited proteinase activities. Majority of the isolates were capable of forming mature biofilms. Interestingly, the presence of serum distorted the yeast sensitivity to fluconazole, but not amphotericin B. Taken together, our findings demonstrate that CBI isolates of *Candida* have the potential to express to varying extent their virulence attributes (e.g., biofilm formation, hemolysin production, and proteinase activity) and these, together with perturbations in their antifungal sensitivity in the presence of serum, may contribute to treatment complication in candidemia. The effect of serum on antifungal activity warrants further investigations, as it has direct clinical relevance to the treatment outcome in subjects with candidemia.

## Introduction

*Candida* is an opportunistic pathogen that can cause life-threatening systemic and bloodstream infections in humans (Calderone and Clancy, 2002). It is the fourth leading cause of bloodstream infection in the United States, accounting for approximately 9% of the total bloodstream infections, following coagulase-negative *Staphylococci, Staphylococcus aureus*, and *Enterococcus* species (Wisplinghoff et al., 2004). In recent reports, *Candida* spp. remains the leading fungal cause of central line-associated bloodstream infections (Hidron et al., 2008; Sievert et al., 2013). Despite the advent of many new antifungal agents, the incidence of *Candida* bloodstream infection (CBI) has been steady over the past decades (Pfaller and Diekema, 2007). In addition to its high incidence, the attributable mortality rate and the associated cost burden are substantial (Wilson et al., 2002; Warnock, 2007). In Hong Kong, an epidemiological study (Yap et al., 2009) revealed a high prevalence, associated mortality, and morbidity of CBI.

Of the *Candida* species, *Candida albicans* is by far the predominant species of CBI (Pfaller et al., 2001, 2011; Labbé et al., 2009). However, recently, the incidence of CBI caused by non-*albicans* species (NAC) has increased and some of the common species isolated are *Candida tropicalis, C. parapsilosis, C. glabrata, C. guilliermondii, C. dubliniensis*, and *C. krusei* (Falagas et al., 2010). The key virulence factors of *Candida* that are associated with bloodstream infections include hemolysin production, proteinases production and biofilm formation (Calderone and Fonzi, 2001; Lim et al., 2012). Hydrolytic enzymes, such as proteinases, of *Candida* species sequester nitrogen from proteins of the host and facilitates tissue invasion (Staib, 1966; Schaller et al., 2005), whereas, hemolysin is needed to acquire iron from the hosts (Nayak et al., 2013). However, it should be noted that the relevance of secreted aspartyl proteinases to the fungal virulence is questionable as shown in data from animal studies (Correia et al., 2010).

Biofilm formation is another feature that contributes to *Candida* pathogenicity in catheter-related bloodstream infection (Shin et al., 2002). *Candida* biofilm is known to be highly resistant to antifungal agents, and it is thus a key attribute to the mortality in bloodstream infections (Seneviratne et al., 2008a). In addition, rising drug resistance among *Candida* species has posed a great challenge to clinicians, especially when treating bloodstream infections (Pfaller et al., 2011). Furthermore, there are only a few studies in the literature that examine the antifungal susceptibility and virulence attributes of CBI such as biofilm formation in Asian populations (Shin et al., 2002; Seneviratne et al., 2011; Tay et al., 2011; Kaur et al., 2014; Tellapragada et al., 2014).

In general, the pharmacologic effect of protein-bound drugs is lower than their unbound counterparts. The protein binding of a drug influences the amount of free unbound drug at the site of infection, as well as its pharmacokinetics and pharmacodynamics (Ashley et al., 2006). This is particularly important for drugs targeting bloodstream infections where the drug is intrinsically exposed to the serum proteins. However, studies on *Candida* bloodstream isolates rarely attempted to capture the latter, real-life scenario by evaluating the *in vitro* minimum inhibitory concentration (MIC) of antifungals against these isolates in the presence of serum.

In the present study, we comprehensively evaluated 63 isolates from candidemic patients for their pathogenic attributes such as hemolysin and proteinase production, and biofilm formation as well as the susceptibility to the two most commonly used antifungals, amphotericin B (a fungicidal agent) and fluconazole (a fungistatic agent). Moreover, taking the foregoing research gap into consideration, we also evaluated the MIC of these antifungal agents in a serum-laced environment. Our study demonstrated that CBI isolates are able to express pathogenic attributes to varying extent; furthermore, the susceptibility of these isolates against fluconazole is influenced in the presence of serum.

## Materials and methods

### Species identification of *Candida* bloodstream infection isolates

Anonymous archival collection of *Candida* isolates was used in the study with the approval of exemption from the Institutional Review Board of the University of Hong Kong/Hospital Authority Hong Kong West Cluster (HKU/HA HKW IRB). It has been accepted by the funding authority, the Research Office of the Food and Health Bureau, the Government of the Hong Kong Special Administrative Region (Health & Medical Research Fund, Project no.: 12111512). This study included 63 CBI isolates derived from two hospitals i.e., Queen Mary Hospital (23 isolates) and Queen Elizabeth Hospital (40 isolates) in Hong Kong. The *Candida* strains were isolated from patients before any antifungal medication was administered. Species identification of *Candida* isolates was performed by two standard culture-dependent methods, namely CHROMagar (CHROMagar™ *Candida*) and commercially available identification kit API 32C AUX method (bioMérieux SA, France; Odds and Bernaerts, 1994). In brief, CHROMagar differentiates various species of *Candida* by formation of specific colored colonies when incubated at 37°C for 48–72 h. API 32C AUX assay is a carbohydrate assimilation test which identifies the species based on their sugar metabolism.

### Antifungal susceptibility testing

Antifungal susceptibility testing of the CBI isolates in planktonic mode was performed using Clinical Laboratory Standards Institute method (CLSI) protocol M27-A3 (broth microdilution assay; Seneviratne et al., 2008b; Fothergill, 2012). Two-fold dilution series of amphotericin B and fluconazole was prepared in RPMI 1640 medium. For the serum induction experiment, RPMI 1640 supplemented with 50% (v/v) human serum (Sigma) was used (Wiederhold et al., 2007). Inocula from 24 h *Candida* cultures were harvested and suspended in RPMI with turbidity equivalent to McFarland standard 0.5 (1 × 10^6^ cells/ml) and then diluted to approximately 0.5 × 10^3^–2.5 × 10^3^ cells/ml. The test was performed in pre-sterilized, flat-bottom 96-well polystrene plates (Iwaki, Japan). *C. albicans* ATCC 90028 was used as quality control strain. Plates were incubated at 37°C for 48 h. MIC was defined as the lowest concentration of the drug that completely inhibits the growth according to the CLSI criteria.

### Hemolysin assay

Hemolysin assay for *Candida* strains was performed according to a previously validated protocol by our group (Luo et al., 2001). In brief, Sabouraud dextrose agar supplemented with 7% sheep blood and 3% glucose was used to determine the hemolysin production by the CBI isolates. Suspension of yeast (1 × 10^8^ cells/ml) was prepared in phosphate buffered saline (PBS; pH 7.2, 0.1 M) and 10 μl was spot-inoculated on sheep blood agar plates, incubated at 37°C in 5% CO_2_ for 48 h. The diameters of the colony and the transparent halo were measured by computerized image analyzer (Qwin, Leica, UK). The hemolysin index (Hi) was calculated by dividing the diameters of the colony and the transparent halo. The assay was performed on two separate occasions as quadruplicates for all isolates.

### Proteinase assay

The activity of secreted aspartyl proteinases was determined by the bovine serum albumin (BSA) plate assay with some modifications to the previous methods (Staib, 1966; Wu et al., 1996). Suspensions equivalent to 0.5 McFarland standard (1 × 10^6^ cells/ml) were prepared from 18-h yeast cultured in Sabouraud dextrose agar (SDA) and 10 μl was spotted on 1% BSA plates. The plates were incubated at 37°C for 120 h. *C. albicans* ATCC 90028 and *C. parapsilosis* ATCC 22019 were used as positive and negative controls. The plates were stained with staining solution containing 1.25% of naphthalene black in 90% methanol/water (v/v) for 5 min and decolorized in 90% methanol/water (v/v) for 48 h. The diameters of the colony and the transparent halo were measured using the computerized image analyzer (Qwin, Leica, UK). Proteinase production index (Ppr) was calculated by dividing the diameters of the transparent halo and the colony by the diameter of the colony. The assay was performed on two separate occasions as quadruplicates for all isolates.

### Biofilm formation and XTT reduction assay

Biofilm formation of CBI isolates was analyzed by previously validated method by our group (Seneviratne et al., 2008b). In brief, a loopful of 18 h culture grown at 37°C in SDA was harvested and suspended overnight in yeast nitrogen base medium (YNB) supplemented with 50 mM glucose in a rotary shaker at 80 rpm overnight at 37°C. Yeast cells in the late exponential phase of growth were extracted and washed twice with PBS. Then, the cells were re-suspended in YNB supplemented with 100 mM glucose with turbidity equivalent to 4 McFarland standard. *C. albicans* ATCC 90028 was used as a control for comparison. Hundred microliters of the yeast suspension was transferred to the 96-well polystrene plate and incubated at 37°C for 90 min (adhesion phase) in an orbital shaker rotating at 80 rpm. Then, the medium was aspirated and the biofilms were washed twice with 100 μl of PBS to remove unattached cells. After washing, 200 μl of YNB medium with 100 mM glucose was added to each well. The plates were incubated at 37°C in a rotary shaker at 80 rpm for 48 h, with a change of the growth medium at 24 h. After the 48 h incubation period, the growth medium was pipetted out and the biofilms were washed twice with 200 μl of PBS before quantifying with XTT reduction assay (Ramage et al., 2001). In brief, 200 μl of the XTT solution was added to the wells and the plate was incubated in the dark at 37°C for 3 h. The XTT solution consisted of 40 μl of XTT stock solution (1 mg/ml in PBS) and 2 μl of menadione (0.4 mM in acetone) topped up to 200 μl in PBS. After incubation, 100 μl of the colored solution was aspirated from all the wells, transferred to Eppendorf tubes and centrifuged at 8000 rpm for 10 min. The centrifuged solution was transferred to a different microtitre plate and the optical density (OD) of the change in color was measured using a plate reader (SpectraMAX 340 Tunable Microplate Reader; Molecular Devices Ltd., Sunnyvale, CA) at 490 nm. This test was performed in duplicates.

### Genotyping of the *Candida* isolates by random amplification of polymorphic DNA (RAPD)

The genetic similarities of the *C. albicans* and *C. tropicalis* isolates were examined by DNA fingerprinting through RAPD analysis. Genomic DNA of the isolates was extracted using the QIAamp DNA Mini Kit (Qiagen, Germany) according to the instructions of the manufacturer. The PCR master mix was prepared with 2 μL (100 ng/μL) of genomic DNA, 5 μL 10X PCR buffer (200 mM Tris/HCl, pH 8.4, 500mM KCl), 200 μM dNTPs, 25 mM MgCl2, 1 μM primer (T3B, 5′-AGG TCG CGG GTT CGA ATC C-3′; Thanos et al., 1996) and 1.5U Taq Polymerase (Invitrogen). PCR was performed by a thermal cycler (GeneAmp PCR System 9700, Applied Biosystems), with the first five cycles at 94°C for 5 min, followed by 35 cycles of denaturation (94°C, 30 s), annealing (52°C, 2 min) and elongation (72°C, 2 min), and lastly, final elongation at 72°C for 10 min. Positive control (genomic DNA of *C. albicans* SC5314) and negative control (water) were added in each PCR run. Gel electrophoresis of the PCR products was performed in 1% agarose gel at constant voltage of 150V for approximately 1 h. The bands were visualized by UV light (ChemiDoc Imaging System, Bio-Rad, USA) after staining with ethidium bromide. The bands of the isolates were analyzed and dendrogram was constructed by the unweighted pair group method in the program GelJ (Heras et al., 2015).

### Statistical analysis

One-way ANOVA with Bonferroni's corrections were used for multiple comparisons of hemolysin index, proteinase production index and optical densities of the XTT reduction assay in Prism 6 (GraphPad Software, La Jolla, CA). A *p*-value of 0.05 or lower was considered to be significant.

## Results

### Species distribution of the isolates

Of the 63 *Candida* bloodstream isolates included in the study, *C. albicans* was the most commonly detected species (*n* = 40), followed by *C. tropicalis* (*n* = 10), *C. parapsilosis* (*n* = 9), *C. glabrata* (*n* = 2), *C. guilliermondii* (*n* = 1) and *C. dubliniensis* (*n* = 1; Table 1).

**Table 1:** **Species distribution of the *Candida* bloodstream infection isolates**.

**Species**	**No. of isolates (%)**
*C. albicans*	40 (63.5)
*C. tropicalis*	10 (15.9)
*C. parapsilosis*	9 (14.3)
*C. glabrata*	2 (3.2)
*C. guilliermondii*	1 (1.6)
*C. dubliniensis*	1 (1.6)
Total	63 (100)

### Antifungal susceptibility testing

The planktonic cells of all the isolates were susceptible to amphotericin B, except for a single isolate of *C. tropicalis* exhibiting marginal resistance with 2 μg/ml as MIC (Table 2). A total of 31.7% of the CBI isolates was resistant to fluconazole (MIC > 32 μg/ml). Of the *C. albicans* isolates, 16 (40%) were resistant to fluconazole. For the NAC, all the *C. glabrata* isolates were resistant to fluconazole, whilst all the *C. guilliermondii* and *C. dubliniensis* were susceptible. However, it has to be aware that the low number of isolates of these three species may not be representative.

**Table 2:** **Antifungal susceptibility of *Candida* bloodstream isolates**.

**Species**	**No. of isolates**	**Amphotericin B**		**Fluconazole**	
		**Susceptible MIC < 2 μg/ml**	**Resistant MIC ≥ 2 μg/ml**	**Susceptible MIC ≤ 32 μg/ml**	**Resistant MIC > 32 μg/ml**
*C. albicans*	40	40 (100%)	0 (0%)	24 (60%)	16 (40%)
*C. tropicalis*	10	9 (90%)	1 (10%)	9 (90%)	1 (10%)
*C. parapsilosis*	9	9 (100%)	0 (0%)	8 (88.9%)	1 (11.1%)
*C. glabrata*	2	2 (100%)	0 (0%)	0 (0%)	2 (100%)
*C. guilliermondii*	1	1 (100%)	0 (0%)	1 (100%)	0 (0%)
*C. dubliniensis*	1	1 (100%)	0 (0%)	1 (100%)	0 (0%)
Total	63	62 (98.4%)	1 (1.6%)	43 (68.3%)	20 (31.7%)

MIC, minimum inhibitory concentration.

Interestingly, serum-laced AST media did not alter the activity of amphotericin B. On the other hand, 9 out of 63 isolates showed an increase in MIC of fluconazole in serum-laced media (Table 3). Seven isolates exhibited four-fold raise in MIC (S18, S25, M4, M5, M6, M8, and M16) and two isolates exhibited three-fold increase (S29 and S14). On the contrary, a few isolates (S15, S17, S36, and S11) showed three-fold reduction in the MIC in the serum-laced medium (Table 4).

**Table 3:** **Fluconazole susceptibility of *Candida* bloodstream isolates that showed increase in MIC under 50% serum induction**.

**Isolates**	**MIC (μg/ml)**	
	**RPMI**	**RPMI + 50% serum**
***C. albicans*^a^**		
S18	8	128
S25	8	128
S29	16	128
M4	4	64
M5	2	32
M6	2	32
M8	8	128
***C. tropicalis*^b^**		
S14	8	64
M16	8	128

MIC, minimum inhibitory concentration.

a 17.5% (7 out of 40) of C. albicans isolates.

b 20% (2 out of 10) of C. tropicalis isolates.

**Table 4:** **Fluconazole susceptibility of *Candida* blood isolates that showed decrease in MIC under 50% serum induction**.

**Isolates**	**MIC (μg/ml)**	
	**RPMI**	**RPMI + 50% serum**
***C. albicans*^a^**		
S15	16	2
S17	32	4
***C. glabrata*^b^**		
S36	32	4
***C. tropicalis*^c^**		
S11	32	4

MIC, minimum inhibitory concentration.

a 5% (2 out of 40) of C. albicans isolates.

b 50% (1 out of 2) of C. glabrata isolates.

c 10% (1 out of 10) of C. tropicalis isolates.

### Hemolysin activity

The mean hemolysin index of the *C. albicans* isolates was the lowest among all the species tested (1.592 ± 0.129). It was significantly lower than the mean hemolysin index of *C. tropicalis* and *C. glabrata*. Only two out of nine *C. parapsilosis* isolates produced hemolysin on the blood agar, while all isolates of other species exhibited hemolytic activity (Table 5).

**Table 5:** **Hemolysin index and proteinase index of the *Candida* bloodstream infection isolates**.

**Species (n)**	**Hemolysin index mean ± SD**	**No. of hemolysin-positive isolate/total no. of isolates**	**Proteinase index mean ± SD**	**No. of proteinase-positive isolate/total no. of isolates**
*C. albicans* (40)	1.592 ± 0.129	40/40	1.854 ± 0.262	31/40
*C. tropicalis* (10)	1.949 ± 0.206^a^	10/10	1.799 ± 0.130	8/10
*C. parapsilosis* (9)	1.778 ± 0.230	2/9	1.640 ± 0.101	6/9
*C. glabrata* (2)	2.058 ± 0.078^a^	2/2	1	0/1
*C. guilliermondii* (1)	1.727	1/1	1	0/1
*C. dubliniensis* (1)	2.074	1/1	1	0/1

n, number of isolates; SD, standard deviation; a, significant higher than C. albicans. The hemolysin index was calculated by dividing the diameter of the transparent hemolytic halo with that of the fungal colony. Similarly, the proteinase index was determined by dividing the diameter of the transparent proteolytic halo with that of the fungal colony. A value of one indicates the absence of enzyme activity, and is excluded in the calculation of the mean. The hemolysin and proteinase indices of each of the individual isolates are provided in Supplementary Table 1.

### Proteinase activity

No proteinase activity was observed in the *C. glabrata, C. guilliermondii*, and *C. dubliniensis* isolates (Table 5). Proteinase activity was observed among the remaining species (*C. albicans, C. tropicalis*, and *C. parapsilosis*) and no statistical significant difference was observed between the mean proteinase indices of these three species.

### Biofilm formation and XTT assay

**Figure 1:**
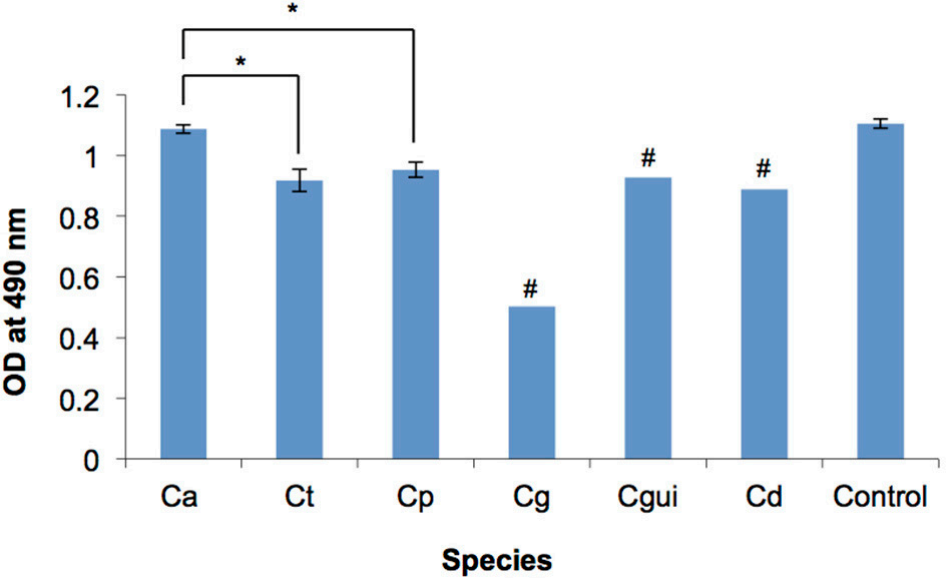
**Biofilm formation of the *Candida* bloodstream infection isolates measured by XTT reduction assay**. OD, optical density; Ca, *C. albicans*; Ct, *C. tropicalis*; Cp, *C. parapsilosis*; Cg, *C. glabrata*; Cgui, *C. guillermondii*; Cd, *C. dubliniensis*; Control, *C. albicans* ATCC 90028; Error bars, standard deviation; ^#^ Standard deviations could not be determined due to the low number of isolates, ^*^p < 0.05. The biofilm of each *Candida* bloodstream infection isolate was quantified by XTT reduction assay. The readings of isolates of each *Candida* species were averaged. Among all the *Candida* species tested, *C. albicans* biofilm was the most robust, whilst *C. glabrata* biofilm was the least robust. *C. albicans* biofilm was significantly more robust than those of *C. tropicalis* and *C. parapsilosis*. No significant difference was observed between the biofilm of *C. tropicalis* and *C. parapsilosis*. The optical density of each of the individual isolates is provided in Supplementary Table 1.

*C. albicans* formed significantly more robust biofilms when compared to NAC (Figure 1). Of the two *C. glabrata* isolates, one produced very minimal biofilm, which gave the optical density (OD) of 0.138 as examined by XTT reduction assay and was 10 times less than the average optical density of the *C. albicans* biofilm (OD = 1.087). *C. guilliermondii* and *C. dubliniensis* produced moderate biofilms (OD = 0.9).

### Genotyping of the *Candida* isolates by (RAPD)

Genotyping was performed for C. albicans and C. tropicalis strains. It seemed that strains derived from Queen Mary Hospital (23 isolates) and Queen Elizabeth Hospital (40 isolates) are genetically quite similar (Supplementary Figures 1, 2). There was no clear genotype specially associated with a particular hospital. There was also no clear association between the genotype of the species with their phenotypic features of biofilm formation, hemolysin index and proteinase index.

## Discussion

Candidemia due to NAC has shown a steep rise in recent decades (Samonis et al., 2008; Rodríguez et al., 2010). In the present study, NAC accounted for a high proportion of all the CBI isolates collected (36.5%), of which *C. tropicalis* was the most common. These results reaffirm the findings of ours (Seneviratne et al., 2011) and a 9-year long study conducted by Yap et al. (2009), where NAC accounted for 46% of the 128 CBI isolates collected in Hong Kong, with *C. tropicalis* being the most common NAC. Similarly, *C. tropicalis* is also the most common NAC amongst the *Candida* bloodstream isolates collected in other regions of Asia (Chen et al., 1997, 2011; Jung et al., 2012; Chander et al., 2013; Kaur et al., 2014). These data are in contrast to those from Europe and the Northern and Latin America, where *C. glabrata* and *C. parapsilosis* were the most common NAC in bloodstream isolate (Pfaller et al., 2011). Clinicians should be mindful of the geographical variation in the prevalence of different NAC species, as they are often associated with higher mortality and resistance to antifungals (Pfaller et al., 2011; Silva et al., 2012).

The increased prevalence of fungal infections and the concomitant prescription of antifungals, have led to emergence of drug-resistant *Candida* strains in the communities worldwide (Arendrup et al., 2013). For instance, fluconazole-resistance is now widespread owing to increased use of antifungals (Anaissie et al., 1996; Kanafani and Perfect, 2008). In a previous study we reported that all of the Hong Kong derived CBI isolates (including *C. tropicalis*) were susceptible to amphotericin B and fluconazole (Seneviratne et al., 2011). In contrast, in the present study, almost a third (31.7%) of the CBI isolates were resistant to fluconazole. Indeed, a single isolate of *C. tropicalis* showed marginal resistance (2 μg/ml) to amphotericin B (Table 2). Resistance to amphotericin B has been recorded rarely in the past, especially in *C. tropicalis* (Drutz and Lehrer, 1978). These data point toward a rather insidious emergence of drug-resistance in CBI in Hong Kong, and hence, the need for constant vigilance accompanied by clinical surveillance studies.

Protein binding plays an important role in determining the pharmacodynamics of a drug. Various studies have shown that serum alters the MIC of antifungal drugs (Zhanel et al., 2001; Bekersky et al., 2002). Higher dose is required for highly protein-bound drugs to exhibit the same microbial killing efficiency when compared to low protein-bound drugs (“free drug hypothesis”; Drusano, 2004). Amphotericin B is a highly protein-bound drug (>95%) and it is anticipated that there would be an increase in MICs for *Candida in vitro* in the presence of serum proteins, while the MICs of fluconazole, which is a weakly-bound drug (11%), may remain unchanged (Humphrey et al., 1985; Bekersky et al., 2002; Ashley et al., 2006).

Other studies have shown that half maximal effective concentration (EC50) of amphotericin B significantly increased for *C. albicans* ATCC 90028 and *C. lusitaniae* in RPMI supplemented with 4 and 8% human serum albumin (Lewis et al., 2006). In contrast, some studies exhibited results contradictory to the free drug hypothesis (Zhanel et al., 2001; Zeitlinger et al., 2011; Elefanti et al., 2013). In the study by Zhanel et al. (2001), the MICs of amphotericin B of all the 10 isolates examined were not altered in RPMI with 80% fresh human serum; whereas, 64% of the isolates tested displayed increase in MIC of fluconazole in RPMI with 80% human serum, and the remaining isolates showed no change in MIC. In our study, all the isolated examined displayed no change in the MICs to amphotericin B in the presence of serum proteins. As for fluconazole, the MICs of the majority of the isolates remained unchanged, but 14.3 and 6.3% of the 63 isolates exhibited an increased and decreased MICs, respectively in the presence of serum proteins (Tables 3, 4). Our data, therefore, confirm the notion that the *in vitro* efficacy of an antifungal drug does not necessarily depend upon its protein binding capacity as suggested by others (Zhanel et al., 2001; Elefanti et al., 2013).

Hemolysin is produced by some species of *Candida* which destroy the circulating erythrocytes to acquire elemental iron from hemoglobin (Schaible and Kaufmann, 2004). In the present cohort, all the CBI isolates, except *C. parapsilosis*, exhibited hemolytic activity (Table 5). Interestingly, the hemolytic-positive isolates of NAC species exhibited higher hemolytic activities than *C. albicans.* This is in contrast to the studies of Luo et al. (2001) who reported that *C. albicans* as the most potent hemolytic species.

Secreted aspartyl proteinases of *Candida* are thought to degrade human proteins and provide nitrogen for the fungal growth (Naglik et al., 2003). Only *C. albicans, C. tropicalis*, and *C. parapsilosis*) in the present cohort demonstrated proteolytic activities, whilst *C. glabrata, C. guilliermondii*, and *C. dubliniensis* were devoid of such activity (Table 5).

*Candida* spp. are known to form highly organized biofilms, especially on indwelling catheters and other prosthetic devices (Seneviratne et al., 2008a). Different *Candida* species are also known to have both inter- and intra-species variations in biofilm development (Seneviratne et al., 2008a; Silva et al., 2010). In the present study, all the CBI isolates, except *C. glabrata*, were good biofilm formers, with *C. albicans* being superior to other species, followed by *C. tropicalis* and *C. parapsilosis* (Figure 1). It has been found that the mortality of CBI caused by biofilm-forming *Candida* spp. are higher than those caused by non-biofilm-forming counterparts (Tumbarello et al., 2012). Moreover, non-*albicans Candida* species isolated from bloodstream were found to be higher biofilm formers than those isolated from other sites (Shin et al., 2002). Patients treated with anti-biofilm antifungal agent (caspofungin), which demonstrates anti-biofilm efficacy *in vitro*, were more commonly associated with shorter post-CBI hospitalization than those treated with non-anti-biofilm antifungal agent (fluconazole; Tumbarello et al., 2012). Furthermore, the lower antifungal susceptibility associated with *Candida* biofilm is often implicated in treatment complication (Douglas, 2003; Seneviratne et al., 2008a). Our current finding adds to the evidence that biofilm formation is a major virulence factor that may lead to treatment complication of CBI. Genotyping of the *C. albicans* and *C. tropicalis* strains using RAPD showed that the strains derived from Queen Mary and Queen Elizabeth hospitals in Hong Kong are quite similar. There was no clear pattern of genotypic and phenotypic features of the *Candida* strains. This is possibly due to genetic and environmental relatedness of the strains in a single country. Other studies have shown geographical location is a major factor associated with genetic relatedness (Dassanayake et al., 2006). Therefore, future studies should aim to compare genotype of the *Candida* isolates with other regional countries.

In conclusion, the present study demonstrates that CBI isolates are to varying extents capable of expressing virulence attributes such as biofilm formation, hemolysin production and proteinase activity. *C. albicans* is the predominant pathogenic species in Hong Kong patients, while the proportion of NAC species remains high. Our current findings further demonstrate that *C. tropicalis* is the most common NAC isolated from CBI in Asia. Almost all the isolates we have evaluated are able to form mature biofilms. Antifungal resistance among CBI isolates, particularly for fluconazole is variably demonstrated amongst the isolates, a critical factor that should be borne in mind when managing candidaemic patients for effective care. Finally, this study indicates that the presence of serum may perturb the activity of some antifungal agents, a factor that needs to be considered when prescribing antifungals in candidemias.

## Author contributions

CS, DT, and CL conceived and designed the study. DT and CL collected the isolates. CS, SR, and SW performed all the experiments, analyzed the data and wrote the manuscript. LS and LJ provided general guidance and revised the manuscript.

## Funding

This work was supported by the Health and Medical Research Fund (No. 12111422) of the Hong Kong SAR Government.

### Conflict of interest statement

The authors declare that the research was conducted in the absence of any commercial or financial relationships that could be construed as a potential conflict of interest. The reviewer JW and handling Editor declared their shared affiliation, and the handling Editor states that the process nevertheless met the standards of a fair and objective review.
