# Supplementary material for: Antifungal Susceptibility in Serum and Virulence Determinants of Candida Bloodstream Isolates from Hong Kong
Source: Front Microbiol. 2016 Feb 26;7:216. doi: 10.3389/fmicb.2016.00216 (PMC4767892; doi:10.3389/fmicb.2016.00216)
Supplement: Supplementary file 1 [file Table1.DOCX]

**Supplementary table 1.** Optical density of biofilm XTT reduction assay, hemolysin index and proteinase index of each isolate

|  |  | **Biofilm (XTT)** | | **Hemolysin index** | | **Proteinase index** | |
| --- | --- | --- | --- | --- | --- | --- | --- |
| **Species** | **Isolate** | **Mean** | **SD** | **Mean** | **SD** | **Mean** | **SD** |
| *C. albicans* | S1 | 1.026 | 0.056 | 1.584 | 0.096 | 1.698 | 0.089 |
|  | S2 | 1.068 | 0.049 | 1.553 | 0.093 | 1.868 | 0.110 |
|  | S4 | 1.096 | 0.057 | 1.746 | 0.116 | 1.904 | 0.101 |
|  | S5 | 1.083 | 0.049 | 1.572 | 0.176 | 1.820 | 0.072 |
|  | S6 | 1.055 | 0.035 | 1.619 | 0.054 | 2.074 | 0.188 |
|  | S9 | 1.065 | 0.063 | 1.519 | 0.054 | 2.111 | 0.128 |
|  | S10 | 1.044 | 0.058 | 1.471 | 0.043 | 2.026 | 0.369 |
|  | S12 | 1.078 | 0.058 | 1.655 | 0.109 | 1.650 | 0.075 |
|  | S15 | 1.071 | 0.075 | 1.894 | 0.303 | 1.697 | 0.054 |
|  | S16 | 1.107 | 0.052 | 1.581 | 0.111 | 1.631 | 0.101 |
|  | S17 | 1.125 | 0.068 | 1.630 | 0.143 | 1 | N/A |
|  | S18 | 1.124 | 0.041 | 1.430 | 0.062 | 1.685 | 0.065 |
|  | S19 | 0.959 | 0.096 | 1.557 | 0.083 | 2.662 | 0.226 |
|  | S22 | 1.210 | 0.093 | 1.572 | 0.066 | 1.684 | 0.068 |
|  | S23 | 1.255 | 0.047 | 1.622 | 0.034 | 1 | N/A |
|  | S24 | 1.212 | 0.068 | 1.436 | 0.607 | 1 | N/A |
|  | S25 | 1.253 | 0.092 | 1.752 | 0.159 | 1 | N/A |
|  | S26 | 1.208 | 0.078 | 1.660 | 0.134 | 1.758 | 0.078 |
|  | S27 | 1.238 | 0.029 | 1.630 | 0.134 | 1.621 | 0.063 |
|  | S28 | 1.216 | 0.048 | 1.420 | 0.180 | 1.524 | 0.090 |
|  | S29 | 1.128 | 0.071 | 1.425 | 0.064 | 1.580 | 0.108 |
|  | M2 | 1.050 | 0.085 | 1.507 | 0.087 | 1.790 | 0.113 |
|  | M3 | 1.019 | 0.111 | 1.525 | 0.063 | 1.962 | 0.081 |
|  | M4 | 1.013 | 0.174 | 1.589 | 0.066 | 1 | N/A |
|  | M5 | 1.108 | 0.136 | 1.549 | 0.064 | 1.787 | 0.164 |
|  | M6 | 1.025 | 0.131 | 1.816 | 0.298 | 1.803 | 0.113 |
|  | M7 | 1.038 | 0.105 | 1.808 | 0.113 | 1.639 | 0.083 |
|  | M8 | 0.938 | 0.082 | 1.595 | 0.072 | 1.702 | 0.089 |
|  | M9 | 1.088 | 0.074 | 1.653 | 0.191 | 1.551 | 0.158 |
|  | M10 | 1.041 | 0.071 | 1.474 | 0.266 | 1.840 | 0.091 |
|  | M11 | 1.130 | 0.211 | 1.430 | 0.088 | 1.978 | 0.127 |
|  | M12 | 1.100 | 0.128 | 1.345 | 0.079 | 1 | N/A |
|  | M13 | 1.047 | 0.089 | 1.705 | 0.272 | 1 | N/A |
|  | M14 | 1.097 | 0.028 | 1.491 | 0.190 | 1.647 | 0.059 |
|  | M15 | 1.077 | 0.029 | 1.758 | 0.301 | 1.799 | 0.072 |
|  | M17 | 1.081 | 0.029 | 1.594 | 0.141 | 1 | N/A |
|  | M20 | 0.931 | 0.058 | 1.578 | 0.093 | 1 | N/A |
|  | M21 | 0.950 | 0.030 | 1.498 | 0.090 | 1.953 | 0.103 |
|  | M22 | 0.947 | 0.030 | 1.583 | 0.081 | 2.420 | 0.141 |
|  | M23 | 0.962 | 0.040 | 1.684 | 0.113 | 2.349 | 0.120 |
| *C. tropicalis* | S8 | 0.917 | 0.060 | 2.247 | 0.111 | 1.819 | 0.031 |
|  | S13 | 0.577 | 0.059 | 1.962 | 0.191 | 1 | N/A |
|  | S14 | 1.194 | 0.024 | 1.896 | 0.117 | 1.821 | 0.107 |
|  | S33 | 0.749 | 0.129 | 2.275 | 0.299 | 1.986 | 0.425 |
|  | S34 | 1.012 | 0.055 | 2.085 | 0.188 | 1.896 | 0.334 |
|  | S40 | 0.983 | 0.035 | 1.823 | 0.062 | 1.537 | 0.071 |
|  | M1 | 1.038 | 0.063 | 1.785 | 0.141 | 1.739 | 0.076 |
|  | M16 | 1.050 | 0.024 | 1.847 | 0.082 | 1.799 | 0.053 |
|  | M18 | 0.796 | 0.053 | 1.951 | 0.060 | 1.794 | 0.117 |
|  | M19 | 0.847 | 0.064 | 1.789 | 0.801 | 1 | N/A |
| *C. parapsilosis* | S3 | 0.869 | 0.043 | 1.826 | 0.054 | 1 | N/A |
|  | S7 | 0.952 | 0.104 | 1 | N/A | 1 | N/A |
|  | S11 | 1.031 | 0.072 | 1 | N/A | 1.766 | 0.112 |
|  | S20 | 0.960 | 0.097 | 1.723 | 0.337 | 1.762 | 0.076 |
|  | S21 | 1.100 | 0.082 | 1 | N/A | 1.603 | 0.109 |
|  | S32 | 0.905 | 0.091 | 1 | N/A | 1.529 | 0.093 |
|  | S35 | 0.914 | 0.062 | 1 | N/A | 1 | N/A |
|  | S37 | 0.982 | 0.047 | 1 | N/A | 1.564 | 0.062 |
|  | S38 | 0.870 | 0.057 | 1 | N/A | 1.617 | 0.053 |
| *C. glabrata* | S30 | 0.138 | 0.129 | 2.135 | 0.736 | 1 | N/A |
|  | S36 | 0.870 | 0.019 | 1.980 | 0.119 | 1 | N/A |
| *C. guilliermondii* | S31 | 0.927 | 0.045 | 1.727 | 0.071 | 1 | N/A |
| *C. dubliniensis* | S39 | 0.889 | 0.035 | 2.033 | 0.255 | 1 | N/A |

SD – standard deviation, N/A – not applicable. A value of one indicates the absence of enzymatic activity.
