# Supplementary figures and images for: Antifungal Susceptibility in Serum and Virulence Determinants of Candida Bloodstream Isolates from Hong Kong
Source: Front Microbiol. 2016 Feb 26;7:216. doi: 10.3389/fmicb.2016.00216 (PMC4767892; doi:10.3389/fmicb.2016.00216)

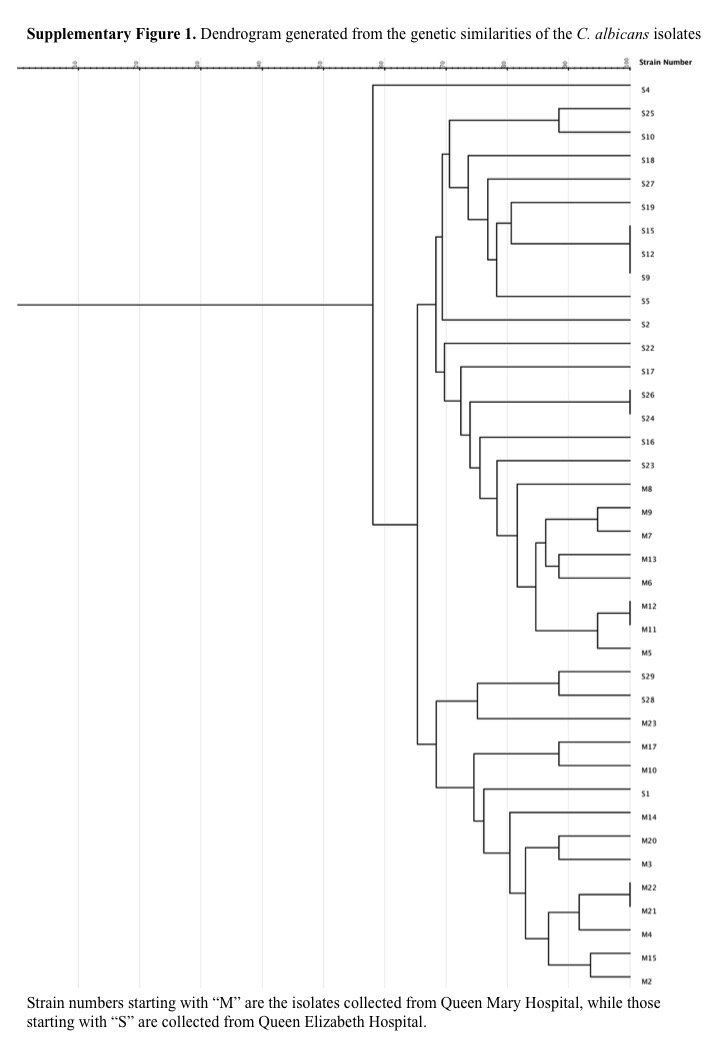

Supplement: Supplementary file 2 [file Image1.JPEG]

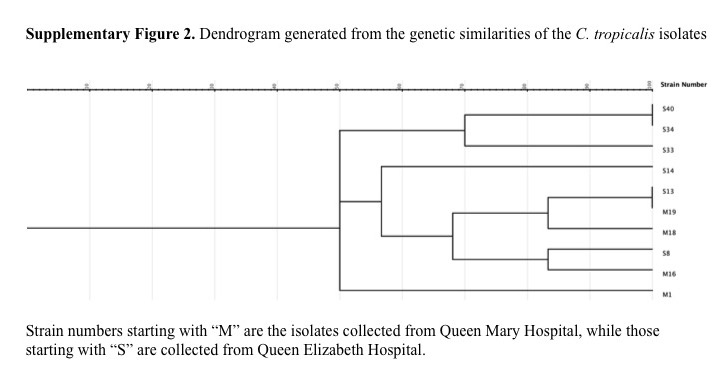

Supplement: Supplementary file 3 [file Image2.JPEG]
